# The Current State of the Art in Autologous Breast Reconstruction: A Review and Modern/Future Approaches

**DOI:** 10.3390/jcm14051543

**Published:** 2025-02-25

**Authors:** Min-Jeong Cho, Michael Schroeder, Jorge Flores Garcia, Abigail Royfman, Andrea Moreira

**Affiliations:** 1 Department of Plastic and Reconstructive Surgery, The Ohio State University Wexner Medical Center, Columbus, OH 43201, USA; min-jeong.cho@osumc.edu (M.-J.C.); michael.schroeder@osumc.edu (M.S.); jorge.floresgarcia@osumc.edu (J.F.G.); 2 Department of Plastic and Reconstructive Surgery, University of Pittsburgh Medical Center, Pittsburgh, PA 15261, USA; abigail.royfman@rockets.utoledo.edu

**Keywords:** autologous, breast reconstruction, microsurgery, DIEP, PAP, APEX flap, four flaps, robotic DIEP, bipedicled flap, flap innervation

## Abstract

**Background/Objectives**: Modern breast reconstruction has undergone substantial evolution, with implant-based, pedicled autologous, and free autologous techniques. The purpose of this study is to review the current state of the art in free autologous breast reconstruction, highlighting advancements in the types of flaps, donor site selection, techniques, and functional restoration. **Methods**: A literature review was conducted using PubMed to capture studies related to well-known free flaps that are used for breast reconstruction. Studies for each flap type were reviewed and sorted for inclusion into one of six categories: (1) clinical outcomes, (2) comparison studies of alternative flaps, (3) preoperative planning, (4) flap classifications and perfusion zones, (5) technique descriptions, and (6) time and cost analyses. **Results**: The majority (77%) of articles included were written on various types of abdominally based free flaps, including TRAM, DIEP, and SIEA flaps. These studies indicated an evolution in technique over time to minimize donor site morbidity, improve patient-reported and functional outcomes, improve efficiency, and expand clinical indications. The remaining 23% of articles discussed alternative flap choices, including PAP, TUG, S/IGAP, and LAP flaps. Studies highlighted technical challenges and the evolution of techniques to make these flaps more accessible, as well as how to combine flaps to expand clinical indications. **Conclusions**: Autologous breast reconstruction has evolved significantly, with advancements in techniques such as robotic-assisted surgery, multi-flap reconstruction, bipedicled flaps, and neurotization. This review highlights the current best practices while acknowledging ongoing challenges and the potential for future innovations in microsurgery, nerve regeneration, and personalized medicine, which hold promise for further refining outcomes.

## 1. Introduction

Breast cancer is the most frequently diagnosed cancer in the United States, affecting approximately one in eight women [1]. Around one-third of breast cancer patients undergo mastectomy as part of their treatment, and 25% of these individuals opt for autologous-based reconstruction [2,3]. Microsurgical breast reconstruction has become the preferred approach for many patients due to its ability to provide a more natural breast appearance, greater long-term durability, and avoidance of implant-related complications [4]. In recent years, the demand for autologous breast reconstruction has surged, driven in part by growing concerns over breast-implant-associated anaplastic large cell lymphoma (BIA-ALCL) and other implant-related illnesses [5,6,7]. These concerns led to a 112% increase in autologous reconstructions between 2009 and 2016 [8,9]. To accommodate this demand, microsurgeons have developed alternative reconstructive techniques for patients who are not candidates for abdominally based procedures due to prior abdominoplasty, limited tissue availability, or personal preference [10,11]. Techniques such as stacked flaps, thigh-based flaps, and trunk-based flaps have expanded the reconstructive possibilities, and advancements are continuing to be made in the field of autologous-based breast reconstruction to this date [12].

As microsurgical breast reconstruction remains the preferred option for women who are interested in a natural result without lifelong monitoring of an implant, especially in those undergoing radiation therapy, it is critical to understand the functional, esthetic, and technical nuances of this procedure and apply algorithmic approaches based on the patient’s needs. Therefore, we performed a review of the literature on this and share technical considerations in modern-day autologous breast reconstruction.

## 2. Materials and Methods

**Figure 1:**
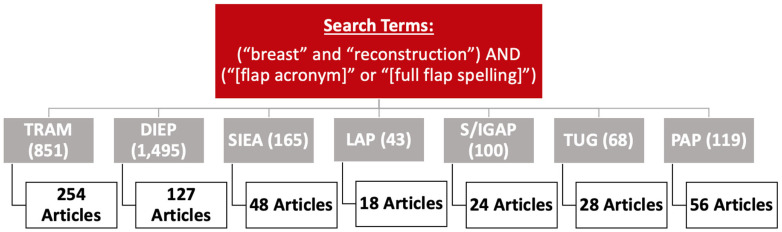
Search algorithm and results.

A review of the literature to characterize the evolution of free autologous breast reconstruction was performed (Figure 1). The search was conducted using PubMed and designed to capture studies related to well-known free flaps that are used for breast reconstruction from January 1980 to April 2024. Flap types included transverse rectus abdominus muscle (TRAM), deep inferior epigastric perforator (DIEP), superficial inferior epigastric artery (SIEA), lumbar artery perforator (LAP), inferior gluteal artery perforator (IGAP), superior gluteal artery perforator (SGAP), transverse upper gracilis (TUG), and profunda artery perforator (PAP) flaps. For each flap type, the search algorithm followed the structure (“breast” and “reconstruction”) AND (“[flap acronym]” or “[full flap spelling]”). For example, the search design for DIEP flap was, (“breast” and “reconstruction”) AND (“DIEP flap” or “deep inferior epigastric perforator”). The inclusion criteria included full-text articles on breast reconstruction using one or more of the free flap types of interest. The exclusion criteria were non-breast-related studies, pedicled flaps, case reports or series that did not describe a new technique, non-human studies, survey studies, literature reviews, non-English texts, and unavailable abstracts.

Abstracts were then reviewed and sorted by one author (M.S.) for inclusion into one of six categories for each flap: (1) clinical outcomes, (2) comparison studies of alternative flaps, (3) preoperative planning, (4) flap classifications and perfusion zones, (5) technique descriptions, and (6) time and cost analyses. Abstracts within each category were then reviewed to identify themes that characterized the evolution of that specific flap over time. Data on techniques, innovations, clinical and patient-reported outcomes, time and cost analyses, and perioperative considerations were extracted from each article. Then, a level of evidence analysis was assigned to each article using the American Society of Plastic Surgeons Level of Evidence rating scale by a separate author (J.F.G.) [13]. Representative articles for each theme were used to create a narrative for each flap from early descriptions to current indications, limitations, and innovations. 

## 3. Results

**Figure 2:**
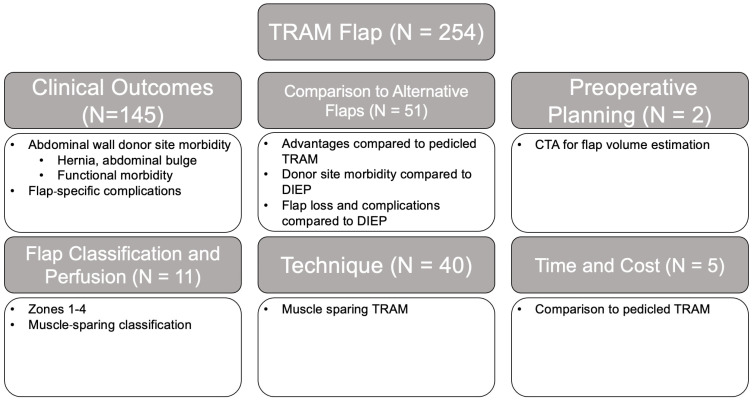
TRAM flap literature review results and associated themes.

**Figure 3:**
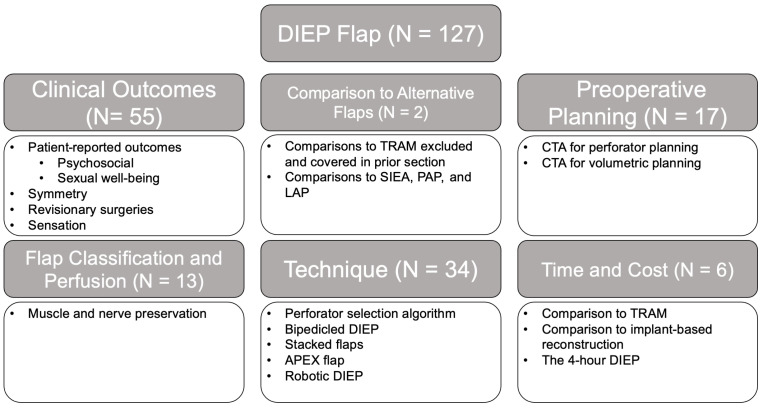
DIEP flap literature review results and associated themes.

**Figure 4:**
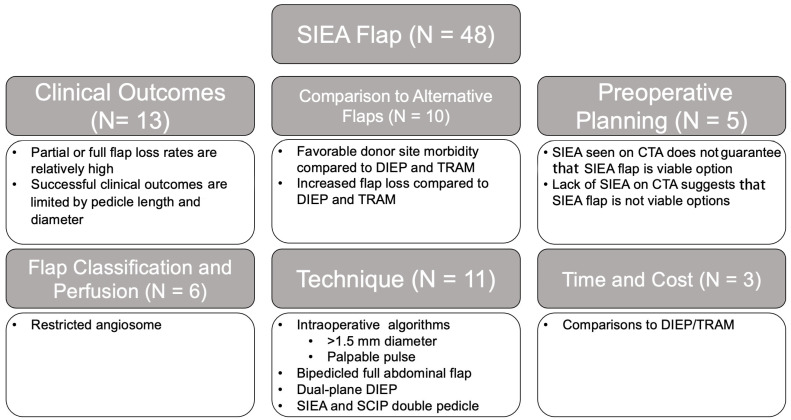
SIEA flap literature review results and associated themes.

**Figure 5:**
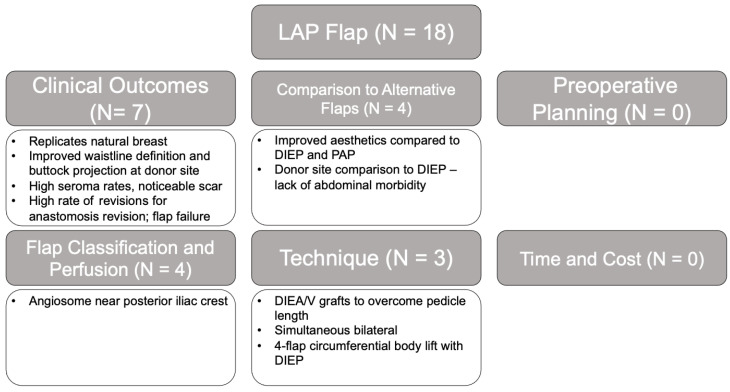
LAP flap literature review results and associated themes.

**Figure 6:**
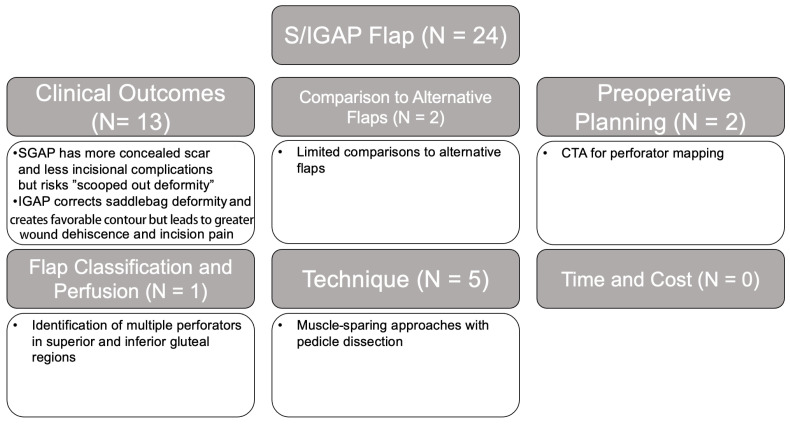
SGAP and IGAP flap literature review results and associated themes.

**Figure 7:**
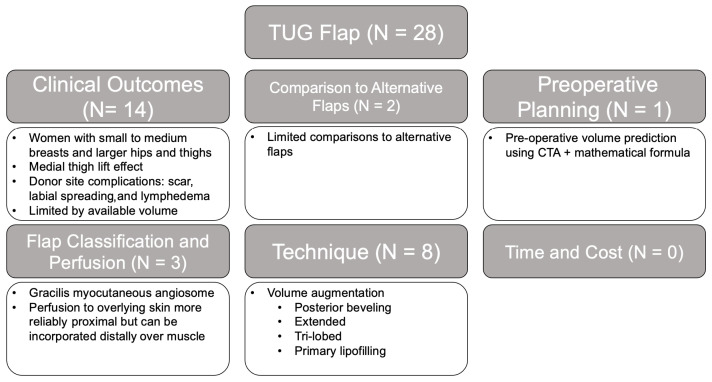
TUG/TMG flap literature review results and associated themes.

**Figure 8:**
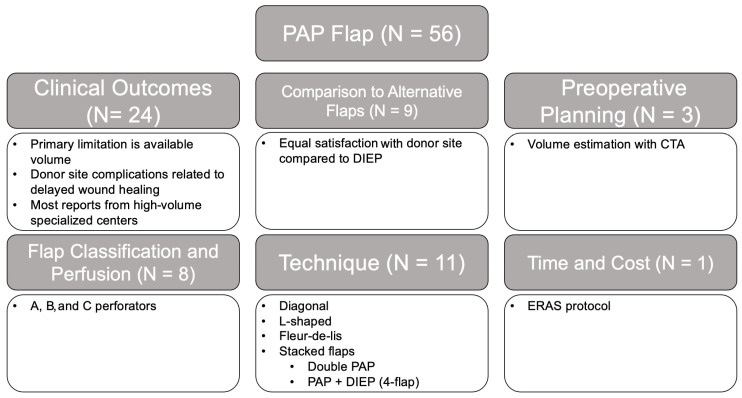
PAP flap literature review results and associated themes.

**Figure 9:**
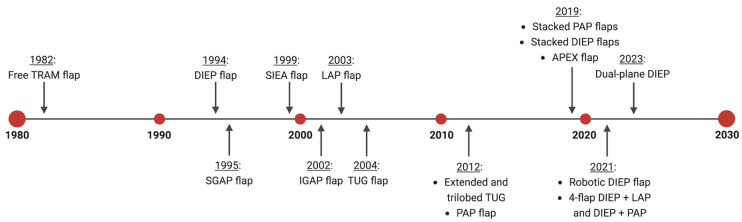
Timeline of the evolution of free autologous breast reconstruction [created using BioRender.com].

The initial search criteria for all flaps yielded 2841 articles: TRAM (851), DIEP (1495), SIEA (165), LAP (43), TUG (68), IGAP/SGAP (100), and PAP (119). After applying the exclusion criteria and screening for relevancy, a total of 555 articles remained: TRAM (254), DIEP (127), SIEA (48), LAP (18), TUG (28), IGAP/SGAP (24), and PAP (56). A breakdown of the number of articles within each category and associated themes for each flap type is provided in Figure 2, Figure 3, Figure 4, Figure 5, Figure 6, Figure 7 and Figure 8. The dates of publication for seminal articles introducing first descriptions of each flap, as well as subsequent articles related to technical modifications and advancements, were used to create a timeline of the evolution of autologous breast reconstruction (Figure 9). Most studies (81.4%) were low evidence levels III (70.6%) and IV (10.8%), with a significantly fewer proportion belonging to levels I (3.1%) and II (15.5%).

### 3.1. Abdominally Based Flaps

Of the 555 studies, 77% (429 articles) were written on various types of abdominally based free flaps such as TRAM, DIEP, or SIEA flaps. The first articles were published in the late 1980s and early 1990s, and high volumes of articles have continued to be published yearly. Of the 429 studies, most were categorized as level III (73.7%), followed by levels II (14%), IV (10.3%), and I (1.6%) (Table 1).

#### 3.1.1. TRAM

The majority of studies on TRAM flaps (57% articles) (Figure 2) primarily focused on clinical outcomes, followed by comparisons to alternative flaps (20%). Most (85%) studies were categorized as low-level evidence, and only 2% of articles were level I. After the establishment of flap vascularity and perfusion in the early 2000s [14], minimizing abdominal wall morbidity quickly became the focus of the clinical outcome studies (23%). As the studies revealed an overall functional decline [15,16] in patients who underwent a TRAM flap procedure, techniques that decrease the abdominal wall morbidity such as muscle-sparing TRAM (ms-TRAM) and DIEP flap [17,18] were popularized and compared with the TRAM flap, accounting for ongoing study over the course of 20 years with mixed results, with some studies suggesting that a greater degree of muscle sacrifice is associated with increased functional morbidity, while other studies suggest similar abdominal bulge and hernia rates in TRAMs when muscle-sparing approaches and/or mesh are used, without any variation related to medial vs. lateral perforator selection [19,20,21,22,23,24,25,26,27]. Flap outcomes have varied by center, with some high-volume centers indicating equivalent flap outcomes between ms-TRAMs and DIEPs [20,28]; other series, as well as a meta-analyses, have suggested a higher rate of major flap complications in DIEPs compared to ms-TRAMs [23,29]. This discrepancy may stem from variations in surgical expertise, patient selection, and institutional protocols. These conflicting outcomes emphasize the need for ongoing, high-quality comparative studies to develop clearer guidelines for flap selection, tailored to patient-specific factors and the surgeon’s experience.

#### 3.1.2. DIEP

Similarly to studies on TRAM flaps, most studies on DIEPs (81.3%) were categorized as having low evidence levels, with fewer belonging to the higher levels I (1.6%) and II (18.1%). However, in contrast to studies on the TRAM flap focusing on the safety of the technique, the studies on the DIEP flap placed importance on evaluating the patient-reported outcomes including esthetics, psychosocial well-being (N = 4), sexual well-being (N = 4), symmetry (N = 6), and sensory loss (N = 5) (Figure 3) [30,31,32,33,34,35,36,37]. As many studies advocated for improved breast, psychosocial, and sexual well-being after undergoing DIEP flap reconstruction [32], subsequent studies evaluated the patient satisfaction rate [34], need for revisionary surgeries, symmetry in unilateral reconstruction [38], and sensation [35].

Thirty-four technique papers on the variations in DIEP flap were identified: abdominal perforator exchange (APEX) flaps (N = 2), stacked DIEP flaps (N = 6), robotic DIEP flaps (N = 3), four-flap DIEP + LAP/PAP flaps (N = 2), dual-plane DIEP (N = 1), and flap neurotization (N = 6). Early outcome studies comparing robotic-assisted versus traditional DIEP flap harvests have shown several advantages of robotic approaches [39,40]. These include shorter fascial incision lengths, a reduced need for abdominal wall reinforcement, and potentially shorter hospital stays [39,41]. While robotic-assisted surgery (RAS) for DIEP flaps tends to take longer than the standard technique, studies have found no significant differences in complication rates [40,41,42]. The extended operative time appears to be balanced by benefits such as less invasive access and improved patient recovery outcomes [30]. As experience with robotic techniques grows, these early findings may further validate the role of robotics in complex flap surgery.

Studies on DIEP flap sensory innervation [43,44,45,46,47,48] have consistently shown that nerve coaptation significantly improves sensory recovery and enhances the quality of life compared to non-innervated flaps. A systematic review by Shiah et al. further confirmed that innervated breasts achieved faster, more consistent, and more uniform sensory recovery across the flap [49]. Despite the heterogeneity in study methodologies, all included studies supported the use of neurotization to enhance sensory outcomes. Although neurotization can extend the operative time, its potential to provide superior sensory recovery makes it a worthwhile consideration in reconstructive surgery. However, further research using standardized sensory testing methods and patient-reported outcome measures is crucial to definitively establish neurotization as the standard of care in breast reconstruction [45,49]. This would help clarify its long-term benefits and refine the technique for broader adoption in clinical practice.

Lastly, there were six studies investigating cost-effectiveness and operative efficiency using preoperative computed tomography angiography (CTA) [50,51,52,53,54] to facilitate perforator selection, volumetric planning, and decision-making [53,54]. The benefits of CTA include detailed vascular mapping, enabling surgeons to select more robust perforators and potentially reducing surgical time. It also allows for a more tailored surgical plan, which is particularly valuable in patients with prior surgeries, helping to reduce complications and improve outcomes. While CTA is not universally mandatory, many surgeons consider it essential for complex cases. In straightforward cases with a clear anatomy or for experienced surgeons using intraoperative Doppler or ultrasound mapping, CTA may be optional. However, for patients with higher BMIs, prior abdominal surgeries, or uncertain vascular anatomies, CTA can be highly beneficial.

#### 3.1.3. SIEA

SIEA flaps (Figure 4) were the least commonly studied among abdominally based flaps (N = 48), and articles on this topic were more evenly distributed among the different categories: clinical outcome (27%), comparative analyses to other flaps (21%), technique variations (23%), preoperative planning (10%), flap classification and perfusion (13%), and time and cost analyses (6%). Again, a large proportion of studies (87.5%) were of low-level evidence: level III (70.8%), IV (16.7%), II (8.3%), and I (4.2%). The majority of studies on the clinical outcome of the SIEA flap found that while the SIEA flap offers favorable donor site function, pain, and esthetics compared to those who have undergone TRAM or DIEP flap procedures [55], it has higher rates of flap-related complications due to a shorter pedicle length, smaller pedicle diameter, and restrictive angiosome [55,56,57,58].

In total, 23% of the studies on the SIEA flap discussed technical considerations of the procedure, including the use of intraoperative algorithms to determine the feasibility of using a SIEA, flap as well as innovative techniques such as the bipedicled full abdominal flap, dual-plane DIEP-SIEA, and SIEA-SCIP double pedicle. Studies recommended using the preoperative CTA to evaluate the caliber of the SIEA vessels and the feasibility of performing variations in the SIEA flap procedure, including a bipedicled flap, SIEA + SCIP flap, and dual-plane DIEP flap [59,60,61,62,63,64]. 

### 3.2. Alternative Flap Choices for Breast Reconstruction

Of the 126 studies on alternative choices for microsurgical breast reconstruction, PAP flaps were the most commonly studied (44%), followed by TUG flaps (22%), S/IGAP flaps (19%), and LAP flaps (14%). Similarly to studies on abdominal flaps, most were categorized as evidence level III (60.3%), followed by II (20.6%), IV (12.7%), and I (6.3%).

#### 3.2.1. LAP

The majority of studies on the LAP flap were on its clinical outcomes (39%), followed by 22% on comparisons to alternative flaps and 17% on technical variations (Figure 5). Due to the novelty of the procedure, the majority of articles were published from two high-volume centers [65,66]. LAP flap studies predominantly consisted of evidence level III (61.5%), had an equal share of evidence levels IV (16.7%) and II (16.7%), and had a single level I study. Clinical studies focused on indications, donor site esthetics, and associated complications, as well as the rate of anastomosis revisions and flap loss. In a high-volume center that offers LAP, DIEP, and PAP flaps for breast reconstruction, LAP flaps were rated as more esthetic compared to DIEP and PAP [66]. LAP flaps avoid dissection of the abdominal wall and create a more defined waistline and projected buttock [67]. However, studies reported relatively high rates of seroma (10–35%), prolonged discomfort and a potentially noticeable scar, and increased technical challenges, including limited pedicle length, multiple intraoperative position changes, and a high rate of anastomotic revision due to the increased number of total anastomoses [68,69].

#### 3.2.2. SGAP and IGAP

Of the 24 studies on S/IGAP flaps, 54% were on clinical outcomes, which examined flap outcomes and donor site differences between SGAP and IGAP, especially on the location of the scar and need for future fat grafting (Figure 6) [70,71]. In addition, 21% of the studies discussed variations in techniques such as muscle-sparing approaches and technical challenges of harvesting flaps due to a short pedicle length and position changes [72,73,74,75,76,77,78]. Low-evidence-level studies were the most common (70.9%); however, there was a relatively larger proportion of levels II (20.8%) and I (8.3%) studies in comparison to other flap types. 

#### 3.2.3. TUG

A total of 28 studies were identified in this category: 50.0% on clinical outcomes, which characterized ideal indications for the flap, 29% on technical considerations to increase the flap volume, 4% on preoperative CTA planning, 11% on flap classification and perfusion, 7% on comparative analyses, and 0% on time and cost (Figure 7). Due to the inherent challenges of the available tissue in thighs, maximizing flap volumes by using preoperative CTA volume prediction models and volume augmentation techniques, including designing the flap to be more posterior to capture bulkier posterior thigh tissue, undermining and beveling the inferior incision to recruit distal subcutaneous tissue, adding a vertical skin component, and primary lipofilling, were discussed [79,80,81]. Relative to other flap types, TUG flap studies had the highest proportion of level I (10.7%) and II (28.6%) studies and the lowest fraction of low-evidence-level studies: level III (60.7%) and no level IV.

#### 3.2.4. PAP

The majority of studies on the PAP flap focused on clinical outcomes (42.9%), which noted limitations on the available volume, the rates of flap loss, and donor site complications related to wound healing (Figure 8). In addition, studies have shown that patients who have undergone reconstruction with PAP flaps are equally satisfied with the donor site compared to those who have undergone DIEP reconstruction from the abdomen [82]. Studies on techniques (20%) included various flap designs including diagonal, L-shaped, and fleur-de-lis [83,84,85], as well as stacked flaps in the form of double PAP flaps and four-flap PAP + DIEP reconstructions [11,86,87]. Similarly to the TUG flap, special considerations for the PAP flap include the limited flap volume, augmentation of the tissue volume using preoperative CTA [88] and flap design [83,84,85,89], technical challenges [90], and donor site dehiscence [51]. These studies were largely of low-level evidence: level III (58.9%), level IV (21.4%), level II (17.9%), and level I (1.8%).

## 4. Discussion

Advancements in breast surgical oncology and plastic surgery have ushered in a new era in mastectomy procedures, integrating oncological principles with a heightened focus on achieving esthetic outcomes. A crucial aspect of the long-term quality of life in women with breast reconstruction is the extent to which their reconstructed breasts feel like a natural part of their bodies. Prior to advances in and the propagation of microsurgical techniques for free tissue transfer, the options for autologous breast reconstruction were limited to local pedicled flaps until the first free TRAM flap for breast reconstruction in the early 1980s [91,92]. Since then, free autologous breast reconstruction has evolved to provide patient-tailored breast reconstruction, maximizing esthetic and functional restoration (Figure 9).

### 4.1. Donor Site Selection: Abdomen and Alternative Sites

As originally described, the free TRAM sacrificed a significant portion of the abdominal wall, prompting the development of the ms-TRAM [93] and eventually the DIEP flap in 1994 [17,18] and the SIEA flap in 1999 [56] (Table 1). Each flap demonstrated decreasing donor site morbidity, with the ms-TRAM minimizing muscle and fascia loss, the DIEP avoiding muscle and fascia loss via meticulous dissection, and the SIEA bypassing muscle and fascia dissection altogether. The DIEP has become the gold standard of abdominally based breast reconstruction, although the ms-TRAM remains a viable option depending on the perforator anatomy and surgeon’s comfort level with intramuscular dissection. Widespread adoption of the SIEA flap has been limited due to its restricted angiosome, which does not cross the midline, and higher rates of flap failure, although the latter may be mitigated by only using the SIEA flap when the pedicle diameter is >1.5 mm or has a palpable pulse [59,60]. 

**Figure 10:**
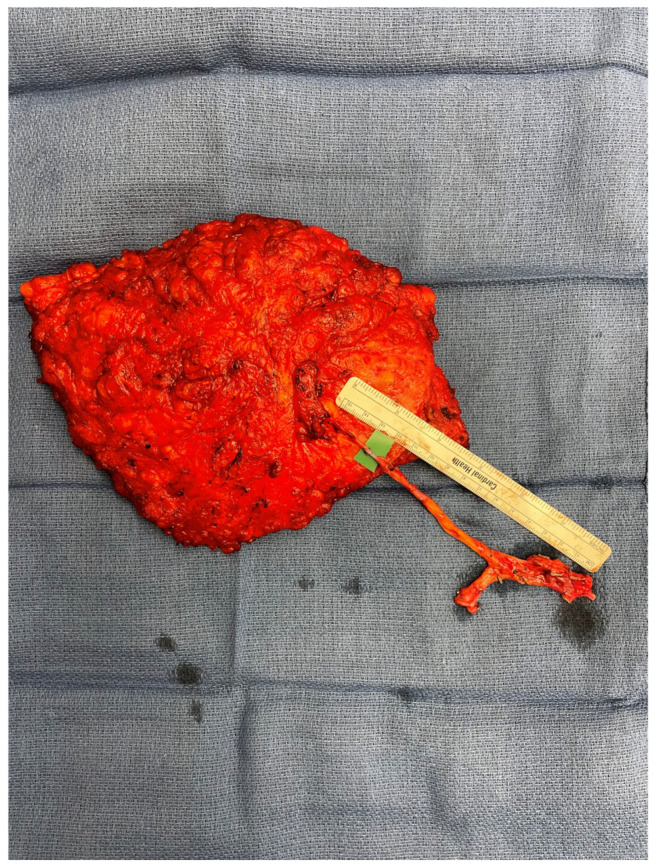
LAP flap with anastomosis to interposition graft.

Our literature review identified the majority of articles on free autologous breast reconstruction to be related to abdominally based flaps (90.3%). However, the early 2010s marked the introduction of new techniques in autologous breast reconstruction, with an increasing number of articles focusing on alternative donor sites, such as the flanks and thighs (9.7%). The LAP flap, which is taken from the flank and was first described in 2003, proved to be a favorable abdominal alternative that provides good volume and replicates the feel of a natural breast while simultaneously creating a more defined waistline and projected buttock [65,67,68,69]. The main obstacles to the widespread use of the LAP flap are related to the need for intraoperative position changes and high technicality. The LAP pedicle is short and requires an interposition graft (Figure 10) equating to four anastomoses per side of reconstruction, leading to a nearly 25% rate of return to OR for anastomosis revisions and flap failure rates ranging from 2 to 9% in high-volume centers [65,66,69]. In our literature review, high-volume studies were limited to just two centers, suggesting that although the LAP flap is a favorable reconstructive option for select women who are not candidates for abdominally based options or want to avoid abdominal donor site morbidity, its general widespread use may be limited due to demanding technical challenges [65,66].

For thigh-based autologous breast reconstruction, the TUG flap, which incorporates the gracilis muscle and overlying skin and soft tissue, was first reported for use in breast reconstruction in 2004 and was the first thigh-based free flap that was used for breast reconstruction [94]. This flap is best suited for women with small to medium-sized breasts, larger hips and thighs, and a lack of abdominal donor tissue [94]. To address the limited amount of tissue volume, many studies focused on augmenting the volume of the flap via CTA volume prediction models, extension of the flap, lipofilling, and a posterior design of the flap. Similarly, donor site complications due to the TUG flap require special considerations due to wound dehiscence, secondary surgery for esthetic refinements, labial spreading, and lymphedema [95]. Overall, the TUG flap is an additional alternative to abdominally based flaps but is primarily limited by its bulk and size. However, preoperative imaging and algorithms, along with intraoperative technique modifications, can optimize patient selection and postoperative results. 

Another thigh-based alternative thigh flap is the PAP flap, which is based on profunda artery perforators that travel through the adductor magnus, supplying tissue and skin on the posterior thigh [96]. The PAP flap has similar indications to TUG flaps, as well as the primary limitation of available volume. A number of flap designs have been reported to overcome this challenge, including L-shaped and fleur-de-lis flaps [83,84,85]. Additionally, PAP flaps have been used as “stacked flaps”, either with another PAP flap or an alternative flap to provide additional volume. The PAP flap has become a versatile flap for breast reconstruction that, although it may be limited by its volume, can be altered in design and used in combination with other flaps to provide excellent patient-specific reconstructions and outcomes with favorable donor site morbidity.

Gluteal flaps include the SGAP and IGAP, which were described for breast reconstruction in 1995 and 1997, respectively [97,98]. Although these flaps provide ample tissue, the wide adoption of these flaps seems to be limited due to their technical demands, need for intraoperative position changes, unfavorable donor site, and alternative available sites [76]. However, recent meta-analyses have helped validate their overall safety when used in appropriate settings [70,71].

### 4.2. Technical Advancements

With the technical advances and increased understanding of flap physiologies, reconstructive microsurgeons shifted their focus from proving a breast mound to esthetically pleasing breasts with minimal donor site morbidity, shorter operative time, and preserved function. The APEX flap, described in 2019 by DellaCroce et al., can be used when the perforator anatomy is such that regular dissection would result in significant rectus muscle transection or sacrifice of rectus motor nerves [99]. In this situation, DellaCroce et al. proposed their technique of the abdominal perforator exchange, in which the primary pedicle is divided between the planned perforators and anastomosed to spare both the muscle and nerves [99]. In their study of 194 flaps, 42% met the criteria for conversion to APEX flaps and had similar outcomes to DIEP flaps in regard to flap survival and fat necrosis [99]. 

**Figure 11:**
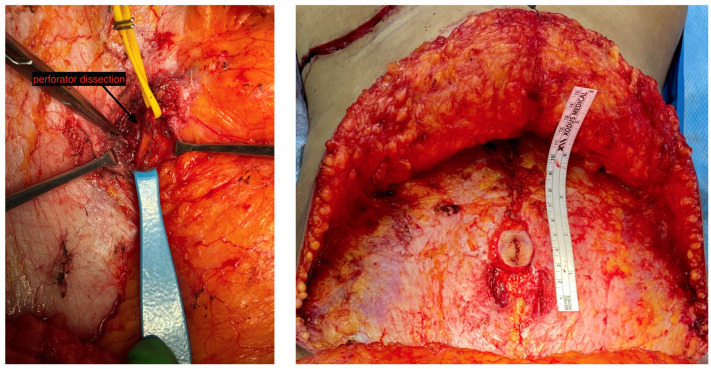
Robotic DIEP dissection (**left**) and limited donor site fascial incision (**right**).

To further address donor site concerns, minimally invasive approaches have emerged. In 2017, Hivelin et al. introduced laparoscopic DIEP flap harvest, utilizing a total extraperitoneal approach [100]. Subsequently, centers have adopted laparoscopy or robotic-assisted methods, with options including TEP or transabdominal pre-peritoneal (TAPP) approaches [40]. The advantages of robotic harvest include a decreased fascial incision length (Figure 11), the elimination of the need for abdominal wall reinforcement, and a potentially reduced hospital stay, without significantly increasing the operative time compared to traditional flap elevation techniques [39]. Indocyanine green-guided near-infrared fluorescence imaging can also be used to guide intra-abdominal dissection of the DIE pedicle to enhance precision and speed and reduce the operative time [101]. The adoption of minimally invasive robotic-assisted techniques, coupled with advanced imaging modalities like indocyanine green-guided near-infrared fluorescence, represents a cutting-edge approach in enhancing the safety, precision, and patient outcomes in autologous breast reconstruction through DIEP flap harvest.

### 4.3. Emergence of Multiple Free Flap Breast Reconstructions

**Figure 12:**
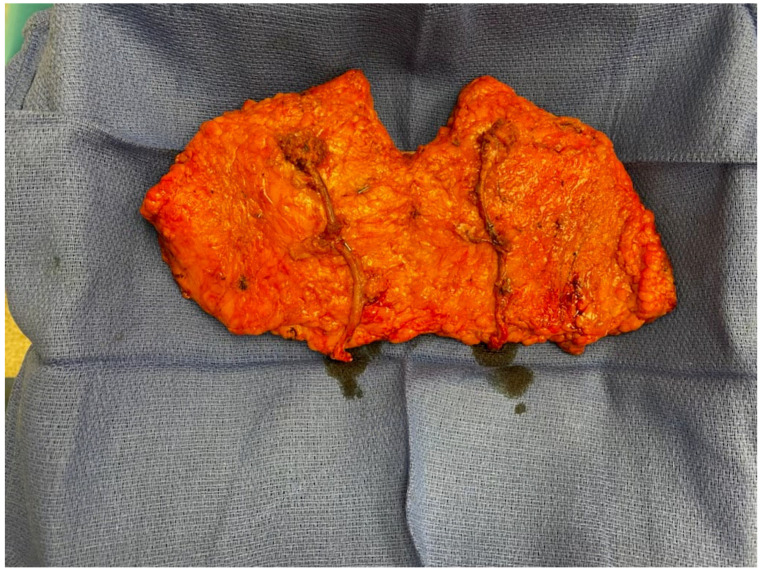
Bipedicled DIEP flap.

In order to address the differences in patients’ available donor sites and their desire for total autologous-based breast reconstruction, a new era of combining flaps has gained traction starting in the 2010s and 2020s to provide women with a wider variety of autologous options for achieving their desired volume. Flaps can be combined by either using a “bipedicled” approach or a “stacked” approach. In the bipedicled DIEP approach, the entire lower abdomen is raised as one flap with both deep inferior epigastric arteries (Figure 12), and multiple anastomotic configurations exist—either anterograde/anterograde or intraflap anastomoses [52]. Similarly, the SIEA system can be leveraged to create a bipedicled flap and planned using preoperative CTA criteria [52] or utilized as a dual-plane DIEP flap, whereby the cranial extent of a DIEA/V perforator is anastomosed to the SIEA/V, which can be used to minimize flap failure and fat necrosis in settings of superficial dominance or to augment the lateral zones of abdominal free flaps [64]. These techniques provide women undergoing unilateral breast reconstruction with a large ptotic contralateral breast and inadequate hemiabdominal donor tissue an autologous option that otherwise would not be replicable by a single flap or implant-based reconstruction. 

Similarly, stacked flaps further expand the autologous indications for women desiring greater volume than available at a single donor site. Whereas bipedicled flaps provide additional volume from a single donor site, stacked flaps can utilize flaps from multiple donor sites and anastomose them to the recipient vessels through cranial/caudal or parasitic approaches. Different combinations of stacked flaps include PAP + PAP, DIEP + DIEP, DIEP + PAP, and DIEP + LAP. PAP + PAP allows for thinner women undergoing unilateral reconstruction with inadequate abdominal tissue to undergo autologous reconstruction, when otherwise, implant-based reconstruction would be their only option [86,87]. DIEP + DIEP can be used in similarly thin women or for women desiring to match a large contralateral breast [87].

Lastly, four-flap reconstruction, either in the form of bilateral DIEP + PAP or DIEP + LAP, further expands the possibilities for matching the desired volume by providing the maximal available autologous tissue for reconstruction [87]. Most candidates for four-flap reconstruction are women with large ptotic breasts that require a large amount of skin and volume that could not be adequately replicated with implants or a single flap [87]. The PAP flap is a favorable choice in conjunction with abdominally based free flaps due to its volume and long pedicle, without a need for an intraoperative position change [87]. The DIEP + LAP is perhaps the most technically challenging option available, as it requires raising a technically challenging flap in the LAP, which has a short pedicle length and also requires an intraoperative position change (Haddock 2021) [87,102]. However, this is likely to offer the greatest possible volume and has the secondary benefit of a circumferential body lift. 

### 4.4. Functional Restoration of Breast

Sensory recovery in autologous breast reconstruction comprises two key aspects: preserving superficial sensory branches to the subcutaneous and mastectomy skin through nerve-sparing mastectomy and neurotization of the NAC, achieved via autogenous nerves or allografts. Slezak et al. first introduced the use of autologous reconstruction to address postmastectomy sensation loss in 1992, utilizing a neurotized, pedicled transverse rectus abdominis musculocutaneous flap (TRAM) [103]. This innovation spurred other surgeons to explore techniques for re-innervating deep inferior epigastric artery perforator (DIEP) flaps, showing promising outcomes [43,47]. However, a challenge in re-innervating DIEP flaps is the damage to the traditional nerve that is used for breast flap neurotization, the lateral cutaneous branch of the fourth intercostal nerves, during the mastectomy [44,94]. As a result, routine innervation is often limited due to the lack of an undamaged recipient nerve being readily available [44]. Advancements, such as utilizing the anterior cutaneous branch of the third intercostal nerve as a recipient for coaptation, coupled with progress in the field of microsurgery, as well as the adoption of nerve allografts and conduits, have led to progress in improving the outcomes in innervated breast reconstruction [104,105]. Although DIEP flaps regain progressive spontaneous sensation postoperatively [34], nerve coaptation improves sensory recovery compared to non-innervated flaps, and, most importantly, patients undergoing innervated autologous reconstruction have reported enhanced quality of life compared to those with denervated reconstructions [106]. 

Restoring sensation to the skin following mastectomy is becoming an integral part of the breast reconstruction process. The success of breast neurotization depends on a thorough understanding of the breast’s anatomy, precise surgical techniques, and insights into axonal regeneration. Growing evidence supports improved sensory recovery and patient satisfaction post-neurotization, making it crucial to prioritize the preservation and restoration of sensation in reconstructed breasts when feasible. However, neurotization comes with limitations. It requires collaboration with an oncologic breast surgeon who is skilled in preserving the intercostal nerves, and it adds additional operating time. Furthermore, the outcomes can vary based on individual patient factors, such as the extent of nerve damage during mastectomy, the quality of the nerves that are available for coaptation, and variability in the rate of nerve regeneration. While promising, these techniques are not universally applicable, and more research is needed to establish standardized protocols and long-term efficacy.

### 4.5. Future Perspectives 

Over the past several decades, the landscape of autologous breast reconstruction has significantly evolved due to an increased understanding of flap physiology and microsurgical advancements [107]. The TRAM flap, once the gold standard for breast reconstruction, has been largely replaced by the DIEP flap due to the ability to preserve the rectus abdominus muscle, thereby reducing donor site complications [108]. Subsequently, newer types of autologous-based reconstruction such as PAP and LAP flaps have gained popularity due to their ability to provide esthetically favorable outcomes while minimizing donor site morbidity [62,109]. 

While the DIEP flap remains the gold standard for many patients, the PAP flap has continued to gain in popularity as an alternative choice of flap for patients with insufficient abdominal tissue [62]. Similarly, the LAP flap has emerged as a viable option for select patients, particularly those with excess flank tissue, although its technical complexity and need for intraoperative repositioning remain challenges to its broader adoption [109]. In contrast, IGAP/SGAP flaps have lost their popularity over time due to the technical demand, need for position change, and location of the donor site [72,73,74,75,76,77,78].

As the technological advancements continue, robotic-assisted flap harvests and AI-driven preoperative planning may push the envelope in the field of autologous-based breast reconstruction further [39,40,41,42,110,111]. While the integration of robotic surgery and the integration of AI into preoperative imaging are currently limited, their popularity has been steadily increasing. As these technologies continue to develop, they will enable safer, more efficient, and more tailored reconstructive approaches, ultimately enhancing patient satisfaction and long-term results.

### 4.6. Limitations

This study is not without limitations. The limitations of our study include the review, data extraction, and designation of evidence level being performed by separate, single authors (M.S., J.F.G.). Additionally, the predominance of low-level-evidence category III or IV studies, indicating retrospective cohorts and case reports/series, across various flap types limits the generalizability of our findings. While the results of these studies provide valuable insights, a relative lack of level I randomized control trials and meta-analyses limits the clinical decision-making ability. Therefore, future work should strive to conduct larger prospective randomized control trials to enhance the validity of the results. 

## 5. Conclusions

Autologous breast reconstruction has evolved significantly, with advancements in techniques such as the DIEP flap and emerging methods like robotic-assisted surgery and neurotization. At present, no single reconstructive technique can be deemed universally superior to the rest. For many, the DIEP flap remains the gold standard due to its balance of favorable esthetic outcomes and minimized donor site morbidity. However, alternatives such as the PAP and LAP flaps provide options for patients who are poor candidates for DIEP flaps due to inadequate abdominal tissue. With the variety of available autologous-based reconstruction procedures, the choice of breast reconstruction should be tailored based on the individual patient anatomy, surgical expertise, and patient preference. This review highlights the current best practices while acknowledging ongoing challenges, such as minimizing donor site morbidity and optimizing sensory recovery. Future innovations in microsurgery, nerve regeneration, and personalized care hold promise for refining outcomes and further improving patient satisfaction. Continued research and integration of new technologies will be essential to advancing the field.

## Figures and Tables

**Table 1:** Types of autologous-based breast reconstruction.

Flap Type	Indications	Strengths	Weaknesses
TRAM	Sufficient abdominal tissue and patient desires abdomen as the donor site.	Long pedicle length enables anastomosis to recipient vessel	Requires sacrifice of the rectus abdominis muscle
DIEP	Sufficient abdominal tissue and patient desires abdomen as the donor site.	Lowest donor site hernia and bulge incidence amongst techniques transgressing the rectus sheath	Requires variable sacrifice of the rectus abdominis muscle
SIEA	Sufficient abdominal tissue and adequate caliber of the SIEA (typically > 1.5 mm in diameter).	Minimal donor site morbidity due to avoiding violation of the rectus sheath	Use limited by variability of SIEA caliber, unreliable perfusion across midline, and short vascular pedicle
LAP	Secondary option for those who are unsuitable for abdominally based reconstruction with excess lateral trunk.	Potential for improved waistline/buttock contour	Dissection limited to vertebral transverse processes leads to shorter pedicle length—typically requires interpositional grafts from DIE vessels and has high seroma rate
S/IGAP	Secondary option for those who are unsuitable for abdominally based reconstruction. May be primary option in those with smaller breasts and excess buttock tissue, have significant functional demands of the abdomen, or who wish to become pregnant in the future.	Muscle-sparing; firmer tissue that is less likely to create ptosis compared to abdominal tissue	Requires intraoperative position change and can lead to buttock asymmetry following unilateral harvest
TUG	Secondary option for those who are unsuitable for abdominally based reconstruction. May be primary option if patient has adequate thigh tissue or wishes to become pregnant in the future.	Reliable blood supply and has similar tissue pliability to that of breast	Limited to small–moderately sized breast reconstruction and requires muscle sacrifice
PAP	Secondary option for those who are unsuitable for abdominally based reconstruction. May be primary option if patient has adequate posterior thigh tissue or wishes to become pregnant in the future.	Muscle-sparing, can be used in various configurations, and has similar tissue pliability to that of breast	Limited to small–moderately sized breast reconstruction

TRAM = transverse rectus abdominis myocutaneous; DIEP = deep inferior epigastric artery perforator; SIEA = superficial inferior artery; LAP = lumbar artery perforator; S/IGAP = superior/inferior gluteal artery perforator; TUG = transverse upper gracilis; PAP = profunda artery perforator.
